# 范可尼贫血与DNA链间交联损伤修复的研究进展

**DOI:** 10.3760/cma.j.issn.0253-2727.2022.02.019

**Published:** 2022-02

**Authors:** 泓竹 陈, 牛 李, 剑 王

**Affiliations:** 上海交通大学医学院附属上海儿童医学中心遗传分子诊断科，上海 200127 Department of Medical Genetics and Molecular Diagnostic Laboratory, Shanghai Children's Medical Center, School of Medicine, Shanghai Jiao Tong University, Shanghai 200127, China

范可尼贫血（FA）是一组具有较高临床和遗传异质性的罕见先天性骨髓衰竭综合征，其发病率在欧美人群中约为5/100万。FA临床特征主要为儿童期进行性骨髓衰竭、多发性先天畸形（75％的患者可合并多指/趾、小头畸形、皮肤牛奶咖啡斑等异常）以及肿瘤易感等。细胞暴露于各种环境因素、化疗药物或细胞内源性代谢产物后可诱导DNA两条链间产生共价加合物并最终导致DNA链间交联（ICL）的形成[1]–[2]。FA的致病基础是FA通路基因突变导致DNA-ICL修复受阻，从而引起基因组不稳定。患者体内细胞对各种内源、外源性DNA交联诱导剂高度敏感，异常染色体比例增加，大量细胞生长周期停滞在G_2_/M期，并导致细胞生长受阻[3]。FA患者首次诊断年龄跨度较大，从未出生到50岁以上[4]。

一、FA的诊断

自1992年第一个致病基因FANCC被克隆并鉴定以来，目前已发现22个基因的异常可以导致FA表型，包括18个明确致病基因（FANCA、FANCB、FANCC、FANCD1/BRCA2、FANCD2、FANCE、FANCF、FANCG/XRCC9、FANCI、FANCJ/BRIP1、FANCL、FANCN/PALB2、FANCP/SLX4、FANCQ/XPF、FANCT/UBE2T、FANCU/XRCC2、FANCV/MAD2L2、FANCW/RFWD3）和4个FA疑似基因（FANCM、FANCO/RAD51C、FANCR/RAD51、FANCS/BRCA1）[5]。除FANCR为常染色体显性遗传基因，FANCB为X染色体连锁隐性遗传基因外，其余均为常染色体隐性遗传基因。其中FANCA突变最多见，约占60％，其次为FANCC（15％）和FANCG（10％）突变[6]。这些致病基因连同一系列相关调控蛋白，如MHF1、MHF2、FAAP20、FAAP24、FAAP100等，共同组成FA通路[7]，因涉及BRCA1和BRCA2基因，该通路又称为FA/BRCA通路。FA/BRCA通路对DNA-ICL的修复至关重要，其缺陷导致FA患者细胞对DNA交联诱导剂高度敏感，如丝裂霉素C（MMC）、环氧丁烷以及铂类化合物等。最近的研究表明FA患者骨髓衰竭的发生可能源于FA/BRCA通路修复DNA-ICL受阻后导致的造血干细胞分化受阻[8]。

DNA交联诱导剂处理的患者细胞可出现染色体异常增加，典型特征为染色体放射状聚集以及染色体断裂。基于此，目前已开发出染色体断裂试验用于FA的临床诊断。作为FA诊断的“金标准”，该检测方法已在欧美许多国家和地区的临床实验室广泛应用[9]。然而，该方法在我国开展较少，我国快速发展和相对低成本的二代测序很大程度上正在取代传统的细胞遗传学检测。因此，与其他遗传性疾病一样，基因检测在中国FA患者临床诊断中起着至关重要的作用。研究表明中国FA患者致病基因谱与国外患者相似，以FANCA为主，其次为FANCC和FANCG[10]。FANCA基因变异中，外显子大缺失约占20％，这提示多重连接探针扩增技术（MLPA）在FA分子诊断中的重要性。此外，基因芯片技术证实少数患者FANCA基因纯合变异源自16号染色体单亲二倍体。

二、FA的治疗

1. 造血干细胞移植：恢复骨髓造血功能是挽救FA患者生命的关键，异基因造血干细胞移植是目前根治FA骨髓衰竭的唯一方法[11]。但因为细胞存在先天性的DNA损伤修复缺陷，FA患者对常规剂量的环磷酰胺清髓性预处理方案不耐受，需采用降低强度的预处理方案[12]。笔者所在单位前期已完成12例FA患儿的造血干细胞移植治疗，所有患儿均存活，最长随访时间已达85个月[13]。需要注意的是，这类患者移植相关毒性反应（包括放化疗所致物理损伤、移植物抗宿主病、免疫损伤、内分泌疾病等）发生率较高，移植后生存率低于获得性骨髓衰竭性疾病患者，且长期随访发现FA患者移植后罹患肿瘤的风险明显升高[14]。雄激素对部分FA患者骨髓衰竭有一定改善作用，但无法根治，常用于无条件获得合适的异基因造血干细胞移植治疗的患者；此外，长期使用雄激素会引起包括多毛症、肝脏肿瘤以及行为异常在内的多种不良反应[15]。

2. 基因治疗：近年来，伴随载体递送技术和基因编辑技术的快速发展，基因治疗已发展为包括FA在内的儿童遗传性疾病最有前景的治疗手段之一。考虑到约60％的FA患儿为FANCA基因突变，目前已有的研究大多采用分离患者CD34^+^造血干细胞，并在体外借助病毒载体转入有功能的FANCA基因后回输患者体内的治疗策略。为获得足够数量的造血干细胞，治疗时间窗口一般为患者出现骨髓衰竭前[16]。Río等[17]在4例男性FANCA基因缺陷患儿中的研究表明，接受FANCA基因治疗的所有患者的骨髓衰竭均得到了不同程度的纠正，外周血细胞计数以及骨髓CD34^+^细胞在MMC处理后的生存能力均明显上升。最近的随访显示治疗3年后，4例患儿的造血功能仍然处于改善状态，其外周血中持续稳定存在较高比例的FANCA基因纠正细胞[18]。这一治疗策略的关键在于寻找安全高效的病毒载体，鼠白血病病毒载体是较早应用的方法，后出现腺病毒和逆转录病毒等。目前基因治疗研究中主要应用的是慢病毒载体，但也存在潜在的基因毒性[19]。

基因治疗的另一项策略是采用基因编辑技术针对特定变异位点的定点修复。Román-Rodríguez等[20]的研究表明CRISPR/Cas9技术可高效修复患者CD34^+^造血干细胞中的FANCA基因变异，并显著改善细胞增殖能力；小鼠移植模型证实基因编辑后的造血干细胞可纠正骨髓造血衰竭。van de Vrugt等[21]利用CRISPR/Cas9系统在Fancf点突变（c.828insTAAA）小鼠模型中探索了这一方法的应用前景：尽管基因编辑的效率较低（≤6％），但纠正后的小鼠胚胎干细胞的增殖快于未治疗的小鼠细胞，并且MMC作用下的细胞存活率增加了27％。基因编辑的优势在于基因纠正后的造血干细胞及其后代在体内具有很强的选择性优势[22]，但选择合适的编辑位点以及技术至关重要，应尽量避免脱靶效应。

总体而言，相比于异基因造血干细胞移植，自体基因治疗是一种理想的稳定改造基因组的方法，既可降低后续治疗过程中的移植物抗宿主病以及肿瘤风险，也具有高产率和正确率。治疗的关键是在疾病早期收集尽量多的造血干细胞，早期进行干预。尽管已经展示出巨大潜力，但FA的基因治疗探索仍处于起步阶段。此外，病毒载体的安全性、基因编辑技术的脱靶效应以及长期治疗效果评价亦是后续研究需关注的重点。

三、DNA-ICL的修复机制

不同的DNA交联剂可产生不同结构特征的ICL，如MMC多诱导产生5′-CG-3′结构特征的ICL，铂类化合物主要诱导产生鸟嘌呤残基交联（5′-GG-3′、5′-GNG-3′）或鸟嘌呤和腺嘌呤残基交联（5′-AG-3′）特征的ICL，而补骨脂素则更倾向诱导形成由胸腺嘧啶参与（5′-TA-3′或5′-AT-3′）的ICL[23]。不同结构特征ICL的扭曲程度也不尽相同，其修复的难易程度也存在差异。为防止未修复或错误修复ICL引起的染色体重排与基因组不稳定，脊椎动物体内发展出3条修复通路：FA/BRCA、NEIL3和REV1通路，其中以FA/BRCA通路最为重要。

1. FA/BRCA通路：功能上，FA/BRCA通路蛋白分为三大类：FA核心复合物（FANC-A、-B、-C、-E、-F、-G、-L、-M和辅助蛋白FAAP100、FAAP20和FAAP24）、泛素化复合物（FANCD2-I）以及下游修复元件[24]。FA/BRCA通路修复DNA-ICL经历三个阶段：①FANCM识别ICL并作为“桥梁”招募核心复合物其他成员并催化FANCD2-I单泛素化；②泛素化的FANCD2-I招募核酸外切酶（MUS81-FAN1-SLX4-ERCC4）对DNA-ICL进行切割解联；③FA通路对DNA-ICL的这一切除将导致一条染色体单链及其复制产物中产生DNA双链断裂，并进一步由RAD51依赖的同源重组（HR）修复，这一过程还涉及RPA、BRCA1、BRCA2、PALB2以及FANCJ等蛋白的参与[25]–[26]。需要指出的是，脊椎动物细胞还可利用经典的非同源末端连接（c-NHEJ）途径修复DNA双链断裂。两种修复机制有较为严格的细胞周期限定：c-NHEJ主要发生在G_0_/G_1_期，而HR则主要在S/G_2_期发挥修复作用。决定DNA双链断裂修复走向HR途径的关键事件是DNA末端切除：未经末端切除的DNA双链断裂损伤只能由c-NHEJ修复，而一旦经历了末端切除就主要由HR修复。FA/BRCA通路缺陷将导致HR进程受阻，DNA-ICL切割产生的双链断裂转向c-NHEJ完成修复，引起不同染色体之间的连接，进而导致染色体易位的发生以及基因组不稳定[27]。另一条染色体单链及其复制产物上则由交联的残基形成一个单-加合物结构，并进一步由跨越合成途径修复，涉及的聚合酶包括Polκ、Polη和Polν，后续延伸由REV1-Polζ催化[26]。作为最复杂的修复通路，FA/BRCA通路具备修复所有类型DNA-ICL的能力，其修复过程更为精细且准确。

2. NEIL3介导的补骨脂素-ICL修复通路：NEIL3基因位于4q34.3，编码碱基切除修复途径一个重要的DNA糖基化酶，可识别受损或错配的碱基并催化水解糖苷键从而将碱基保持在脱氧核糖主链上[28]。2016年，Semlow等[29]首次将NEIL3与DNA-ICL的修复联系起来。NEIL3可独立于FA/BRCA通路直接修复补骨脂素-ICL，该修复过程不产生DNA双链断裂。NEIL3对ICL的这种切除方式导致一条DNA单链及其复制产物产生一个无碱基空位并通过相应的核酸酶切除修复，另一条DNA单链及其复制产物含有一个损伤的碱基并由REV1依赖的跨越合成途径修复。进一步研究发现，正常情况下约80％的补骨脂素-ICLs由NEIL3修复，其余20％经FA/BRCA通路修复。本课题组近期的研究表明NEIL3可以比FA/BRCA通路蛋白更快被招募到补骨脂素-ICL部位，该过程依赖PARP家族蛋白。通过质谱技术鉴定到NEIL3新的相互作用蛋白RUVBL1/RUVBL2复合物[30]。此外，TRAIP作为一种E3泛素化连接酶，可在上游同时控制NEIL3和FA/BRCA通路对补骨脂素-ICL的修复[31]。至此，一条全新的DNA-ICL修复通路，NEIL3通路被鉴定出来。

3. REV1介导的乙醛-ICL修复通路：机体摄入的酒精在肝脏中被乙醇脱氢酶转化为有毒的乙醛，若后者不能被及时清除将导致DNA-ICL，即乙醛-ICL的生成。乙醛是细胞最主要的内源性DNA交联剂[32]。传统的观点认为乙醛-ICL主要通过经典的FA/BRCA通路修复，如前所述，该过程产生DNA双链断裂并进一步由同源重组修复。然而，Hodskinson等[32]最近的研究发现机体还可利用一个更快速的新修复机制完成对乙醛-ICL的修复，该修复过程依赖于跨损伤合成途径中的DNA聚合酶REV1和polζ等。类似于NEIL3修复补骨脂素-ICL，REV1介导的乙醛-ICL修复通路不涉及DNA链及交联本身的切除步骤，因此不会造成DNA双链断裂。虽然这种修复方式易出错，但避免了DNA链断裂或空碱基位点的产生而导致的染色体重排以及大规模基因组不稳定性[33]。

四、FA/BRCA通路与肿瘤

除介导DNA-ICL的修复外，FA/BRCA通路对维持细胞基因组稳定的重要作用亦体现在不仅参与其他类型DNA损伤的修复（如DNA 双链断裂、DNA复制叉的保护和重启等），并且涉及多种DNA损伤反应信号通路（如ATM/ATR信号通路）[34]。因此，FA患者常面临罹患一系列恶性肿瘤的风险（比正常人高500～700倍），其中以血液肿瘤最为常见[35]，如骨髓增生异常综合征和急性髓系白血病。其发生机制可能源于FA/BRCA通路受阻引起染色体断裂增加，促使细胞凋亡并导致造血干细胞衰竭；为了维持组织内环境的稳定，剩余的造血干细胞反复增殖，引起肿瘤克隆的快速选择，从而推动FA患者血液系统肿瘤发生[36]。据报道，约13％的FA患者在50岁之前会发生急性髓系白血病[37]。此外，实体瘤在FA患者中也较为常见。回顾性研究表明在不考虑年龄的情况下，8.2％～10.5％的FA患者会并发实体瘤，最常见的是头颈部鳞状细胞癌、食道癌、乳腺癌与卵巢癌[38]。FA患者患头颈癌的风险是普通人的200～1000倍，而且发病年龄也更为年轻，其中主要为口腔鳞状细胞癌[39]。

FA致病基因的杂合子携带者是否具有肿瘤高发风险是近年来关注的热点，这一点对FA患者家庭成员以及携带这类基因体细胞杂合突变人群的遗传咨询十分关键。尽管一项基于癌症基因组图谱（TCGA）收录的9种常见癌症基因组分析表明40％的肿瘤存在FA基因突变[40]，目前明确杂合子可导致肿瘤易感的基因仅包括FANCD1/BRCA2、FANCJ/BRIP1、FANCN/PALB2和FANCS/BRCA1[5]。其中，FANCD1/BRCA2、FANCN/PALB2和FANCS/BRCA1是乳腺癌易感基因，FANCJ/BRIP1的杂合突变将增加卵巢癌发生的风险[41]。

此外，FA基因的表观遗传学修饰也可能诱发肿瘤风险。已有研究表明FANCF启动子区域的高甲基化可“灭活”蛋白功能，从而导致肿瘤的发生，包括急性髓系白血病、卵巢癌和宫颈癌、头颈部鳞状细胞癌、非小细胞肺癌、颗粒细胞瘤、乳腺癌和膀胱癌[37],[42]。

需要特别指出的是由于FA患者对链间交联剂敏感及其自身存在的基因组不稳定，这类患者对放化疗耐受性极差、化疗相关毒副作用大，一旦发生肿瘤，可能将面临无药可用的局面。总之，FA患者不仅罹患肿瘤的风险增高，并且在FA/BRCA通路无法正常行使DNA损伤修复功能时，患者细胞本身更容易受到来自抗肿瘤药物的损害[40],[43]。

五、小结和展望

DNA-ICL修复依赖于多个通路共同发挥作用，其中FA/BRCA通路是最主要的途径，以更为精准的方式修复所有类型的交联损伤；而NEIL3和REV1通路可独立于FA/BRCA通路分别特异性地修复补骨脂素-ICL和乙醛ICL。有趣的是，REV1介导的跨越合成途径本身位于FA/BRCA和NEIL3通路的下游。目前，关于NEIL3通路和FA/BRCA通路的关系，以及NEIL3通路是否在FA的发生发展中扮演重要角色仍然未知。阐明细胞如何选择性利用不同的修复通路完成对各类型DNA-ICL的修复对深入理解FA和相关肿瘤的发病机制，并在此基础上开发新的治疗手段具有十分重要的意义。
